# An improved method to quantitate mature plant microRNA in biological matrices using modified periodate treatment and inclusion of internal controls

**DOI:** 10.1371/journal.pone.0175429

**Published:** 2017-04-11

**Authors:** Haiqiu Huang, Jamin Roh, Cindy D. Davis, Thomas T. Y. Wang

**Affiliations:** 1 Diet, Genomics and Immunology Laboratory, Beltsville Human Nutrition Research Center, USDA-ARS, Beltsville, Maryland, United States of America; 2 Office of Dietary Supplements, NIH, Bethesda, Maryland, United States of America; Tianjin University, CHINA

## Abstract

MicroRNAs (miRNAs) ubiquitously exist in microorganisms, plants, and animals, and appear to modulate a wide range of critical biological processes. However, no definitive conclusion has been reached regarding the uptake of exogenous dietary small RNAs into mammalian circulation and organs and cross-kingdom regulation. One of the critical issues is our ability to assess and distinguish the origin of miRNAs. Although periodate oxidation has been used to differentiate mammalian and plant miRNAs, validation of treatment efficiency and the inclusion of proper controls for this method were lacking in previous studies. This study aimed to address: 1) the efficiency of periodate treatment in a plant or mammalian RNA matrix, and 2) the necessity of inclusion of internal controls. We designed and tested spike-in synthetic miRNAs in various plant and mammalian matrices and showed that they can be used as a control for the completion of periodate oxidation. We found that overloading the reaction system with high concentration of RNA resulted in incomplete oxidation of unmethylated miRNA. The abundant miRNAs from soy and corn were analyzed in the plasma, liver, and fecal samples of C57BL/6 mice fed a corn and soy-based chow diet using our improved methodology. The improvement resulted in the elimination of the false positive detection in the liver, and we did not detect plant miRNAs in the mouse plasma or liver samples. In summary, an improved methodology was developed for plant miRNA detection that appears to work well in different sample matrices.

## Introduction

MicroRNAs (miRNAs) are a class of small (21–25 nucleotides), functional, non-protein coding RNAs that ubiquitously exist in microorganisms, plants, and animals, and modulate a wide range of critical biological processes [1]. Experimental and bioinformatic analyses have shown that dysregulation of miRNAs is involved in various diseases, such as inflammation, Alzheimer's disease, and cancer [1–7]. In 2012, Zhang and colleagues reported detectable levels of rice miRNAs (miR156a and miR168a) in human and mouse blood and tissues. These authors proposed the novel concept of cross-kingdom transfer of plant miRNA to human and mouse via dietary intake [8]. In the following years, many studies were carried out to confirm whether dietary small RNAs can be absorbed and detected in mammalian tissues, however, the results have been inconsistent [9–18].

**Fig 1:**
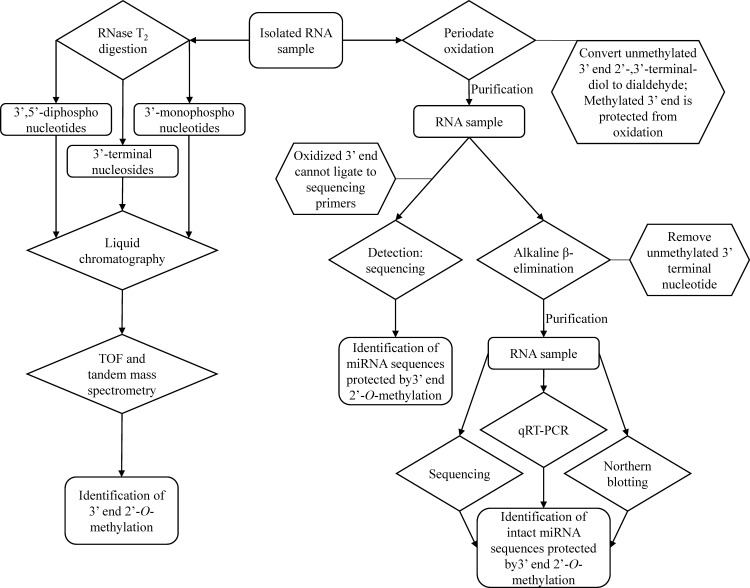
Methods of differentiating mature plant miRNAs from other miRNAs utilizing the 3’ end 2’-O-methylation.

The challenge underlying the discrepancies in the literature may be due in large part to the methodology used for plant miRNA detection. Two critical issues in assessing the origin of miRNAs in human circulation are the difficulty in differentiating 1) mammalian and plant miRNAs and 2) mature and non-mature plant miRNAs. Mature plant miRNAs possess a 2’-*O*-methylation at the 3’ end [19] that stabilizes plant miRNA and provides resistance to RNase and oxidation. Mammalian miRNAs, on the other hand, usually do not have such 3’ end modification [20, 21]. Currently, sequence specificity and 2’-*O*-methylation at the 3’ end of miRNAs are being used as indicators to differentiate the origin of miRNAs. Sequence-specific detection methods, such as sequencing [8, 11], real-time PCR (qRT-PCR) [10, 12, 15], and northern blotting [22], have been used to detect miRNAs (Fig 1). However, these methods cannot differentiate between mature (methylated 3’ end) and non-mature (unmethylated 3’ end) plant miRNAs. The non-mature, unmethylated plant miRNAs, though identical in sequence to the methylated ones, are susceptible to degradation by RNase or oxidation. Therefore, these miRNAs may not be available to exert biological relevant effects upon ingestion.

Mass spectrometry has been used to identify 3’ end 2’-*O*-methylation of plant miRNA by their molecular weight and retention time (Fig 1) [23]. Unfortunately, such detection methods require digesting miRNA into individual nucleotides and nucleosides which destroy the ability to detect specific sequences. More recently, stepwise degradation of polyribonucleotides by periodate oxidation and elimination of unprotected 3’ terminal ribonucleotide has been used to identify 2’-*O*-methylation at the 3’ end [24]. Researchers used periodate oxidation alone or in combination with alkaline β elimination to react with the unmethylated 3’ end of miRNAs, followed by sequencing, qRT-PCR, or gel electrophoresis to detect the product of the reaction (Fig 1) [8, 15, 22]. The combination of periodate treatment and sequencing or qRT-PCR can significantly improve the specificity of plant miRNA detection. Furthermore, mature miRNA-specific stem-loop reverse transcription primers and qRT-PCR were widely used in previous studies to detect and validate the expression level of plant miRNAs [8, 11, 15, 16, 22]. However, validation of periodate treatment or β elimination efficiency and incorporation of proper internal controls in the assay were often lacking. Insufficient reaction time or overloading the reaction system may result in incomplete oxidation and false identification of miRNA origin and amount. Also, variations in recovery of miRNA, in isolation and purification of miRNA, and in the quantitation outside of the dynamic range of qRT-PCR detection can also significantly affect the quantification of miRNA [22, 25]. If these variables are not well controlled, the concentration of miRNAs in biological samples can be under- or over-estimated due to variations in recovery rates or PCR conditions [26]. Hence, critical evaluation of miRNA detection methods and development of a robust analytical protocol are warranted.

Previous studies examined the circulating levels of miRNAs from rice [8], honeysuckle [27], watermelon and fruit juice [22], and other plant sources [10, 15] in mammalian plasma or serum. Corn and soy are among the top four crops produced and consumed in the U.S. and are the most widely used food ingredients in processed foods [28–30]. Profiling studies of corn and soy have identified miR156a, -164a, and -167a as common plant miRNAs which are detected at high levels in corn and soy [31–33]. Therefore, typical human diets would be a good source of these miRNAs [33]. Soybean and rice miRNAs have shown robust stability in an *in vitro* simulated human digestion process [34]. However, a biological study on the digestion and absorption of miRNAs from corn or soy has not been performed. Given the amount regularly consumed by individuals, it would be critical to elucidate the fate of corn or soy miRNAs.

The present study seeks to address critical issues raised above related to analysis of plant miRNA in biological samples and focused on: 1. evaluating the efficiency of periodate treatment to differentiate plant vs. mammalian, mature vs. non-mature miRNAs in different biological matrices, 2. establishing the utility and necessity of including synthetic miRNAs as internal standards, and 3. validating cross-kingdom transfer of miRNA from major plant foods, i.e. corn and soy, into animals. A standardized protocol is presented as a result of this study.

## Materials and methods

### Materials and reagents

Borax, boric acid, sodium hydroxide, sodium periodate, glycerol, sodium acetate (pH 5.2, DEPC-treated) were purchased from Sigma-Aldrich, St. Louis, MO. Glycogen was obtained from Fermentas (part of Thermo Fisher Scientific), Vilnius, Lithuania. Methylated or unmethylated RNA oligonucleotides internal controls were synthesized and purchased from Integrated DNA Technologies (Coralville, IA). Sequences of the RNA oligonucleotides are listed in Table 1.

**Table 1:** Internal control miRNA nucleotide sequences.

miRNA ID	miRNA sequence (5’ to 3’)
miR171j	UAUUGACGCGGUUCAAUUCGA
miR171j_M	UAUUGACGCGGUUCAAUUCGmA *

* m indicates 2’-O-methylation.

### Animals and diets

Male C57BL/6 mice (5-week old, Charles River, Wilmington, MA) were given free access to water and corn/soy-based rodent chow (8728C Teklad Certified Rodent Diet, Harlan Laboratories, Inc, Frederick, MD) for 2 weeks prior to tissue collection. At the end of the feeding period, animals were anesthetized with CO_2_ and sacrificed. Blood was collected by cardiac puncture with syringes previously rinsed with potassium EDTA solution (15% w/v), and plasma was separated by centrifugation at 1500 rpm for 30 min at 4°C. Livers and fecal samples were collected, immediately frozen in liquid nitrogen and kept at -80°C before analysis. This study was carried out in strict accordance with the recommendations in the Guide for the Care and Use of Laboratory Animals of the National Institutes of Health. The protocol was approved by the U.S. Department of Agriculture (USDA) Agricultural Research Service (ARS) Beltsville Area Institutional Animal Care and Use Committee (IACUC) (Protocol # 15–015). CO_2_ euthanasia and other efforts were made to minimize suffering of animals.

### RNA isolation and purification

Soy (Asgrow Soybean Seed, Treatment code: 0, Germination: 85%, Origin: NC) was acquired from Monsanto Company (St. Louis, MO). Corn was purchased from a local grocery store. 30 mg of soy, corn, liver, fecal samples were weighed and homogenized in LiCl extraction buffer using 2 mL Precellys Lysing Kit (Bertin Corp., Villeurbanne, France). RNA was isolated using a method published by Rosas-Cárdenas et al. [35], and then further purified using RNeasy Mini Kit from Qiagen (Cat No.: 74104) following the manufacturer’s protocol. RNA samples were kept at -20°C and used within a week. RNA concentration and purity were determined using Nanodrop 8000 Spectrophotometer (Thermo Fisher Scientific, St. Louis, MO).

### Periodate oxidation

A modified protocol of periodate oxidation was used in this study (see S1 File for the protocol) [8]. A 100 μL mixture consisting of small RNA and nuclease-free water (as a control group) or 10 mM sodium periodate (as treatment group) was incubated at 0°C for 40 min in the dark. 10 mM sodium periodate was added to the control group immediately before the precipitation to achieve the same ion strength between the control and treatment groups. The oxidized RNA was precipitated by ethanol, rinsed twice with 70% ethanol, air dried, dissolved in nuclease-free water, and stored at -20°C and used within a week.

### Periodate oxidation and alkaline β-elimination

A modified protocol of periodate oxidation and alkaline β elimination was used in this study (see S1 File for the protocol) [36]. The specific amount of small RNA was dissolved in 87.5 μL 0.06 M borax/boric acid buffer (pH 8.6), and 12.5 μL of nuclease-free water (as a control group) or 200 mM sodium periodate (as treatment group) were then added. Sodium periodate was freshly prepared before every treatment. Samples were incubated in the dark at room temperature for 1 h. 10 μL of glycerol was added and incubated for another 30 min to stop the reaction. For the control group, 12.5 μL sodium periodate was incubated with glycerol for 60 min prior to mixing with the samples to achieve the same ion strength between the control and treatment groups. 1 μL glycogen, 10 μL 3 M sodium acetate, and 300 μL ethanol was added to precipitate RNA. Then samples were incubated in -20°C for 20 min and centrifuged at 4°C at 14,000 rpm for 10 min. Samples were washed with 70% ethanol twice and air dried. Precipitated RNA was dissolved in 100 μL of 0.055 M borax/boric acid/NaOH (pH 9.5) and incubated for 60 min at 45°C. RNA was then precipitated, washed, and stored at -20°C and used within a week.

### Plant miRNA detection

Plant miRNA was detected using qRT-PCR. TaqMan MicroRNA Assays (Table 2) were purchased from Thermo Fisher Scientific (St. Louis, MO) and used for microRNA reverse transcription and detection. TaqMan MicroRNA Reverse Transcription Kit (Thermo Fisher Scientific, St. Louis, MO) and the small RNA-specific RT primer from the TaqMan MicroRNA Assays were used to reverse transcribe complementary DNA. 5 μL of 2 ng/μL RNA was used in reverse transcription and 1 μL of reverse transcription product was used in qRT-PCR. PCR was performed on ViiA7 qRT-PCR System (Applied Biosystems, Foster City, CA) using TaqMan Universal PCR Master Mix (Cat No.: 4304437) and small RNA-specific TaqMan primer from the TaqMan MicroRNA Assays. The following amplification parameters were used for PCR: 50°C for 2 min, 95°C for 10 min, and 40 cycles of amplification at 95°C for 15 sec and 60°C for 1 min.

**Table 2:** TaqMan MicroRNA Assays.

miRNA	Assay ID	Target miRNA sequence (5’ to 3’)
ath-miR156a	000333	UGACAGAAGAGAGUGAGCAC
ath-miR164a	000344	UGGAGAAGCAGGGCACGUGCA
ath-miR167a	000348	UGAAGCUGCCAGCAUGAUCUA
zma-miR171j	241641-mat	UAUUGACGCGGUUCAAUUCGA

### Statistical analysis

All experiments were conducted in triplicate and representative data was reported as a mean ± standard error. A 0.005 amol to 500 fmol range of synthetic miRNAs were used to construct a standard curve in qRT-PCR. Linear regression and statistical analysis were performed using GraphPad Prism 6 (2015, Graphpad Software, La Jolla, CA). Significance differences between means from treated groups compared to controls were determined using one-way ANOVA and Tukey’s Honestly Significant Difference (HSD) test. Statistical significance was defined at *p* ≤ 0.05.

## Results

### Design of miRNA internal control for plant miRNA detection

**Fig 2:**
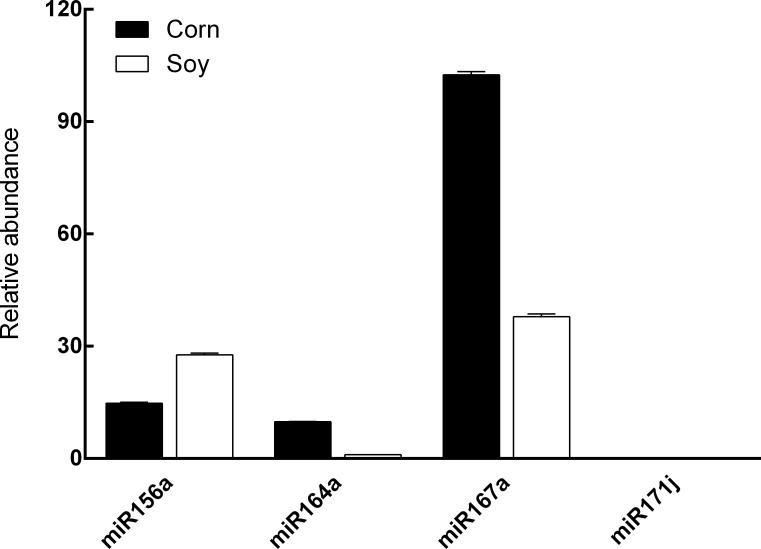
Relative abundance of miR156a, -164a, -167a, and -171j in soy and corn. Expression levels of miRNAs were analyzed by qRT-PCR and relative expression level of miR164a in soy was set as 1 (data presented as Mean ± SD).

First, we considered whether inclusion of an internal control to monitor recovery and efficacy of multi-step treatments is necessary to ensure the validity of the plant miRNA detection assay. The internal control was designed and selected based on two criteria: 1) the specific miRNA should not be present in the material to be examined, and 2) should be usable in PCR-based detection assays that are readily available from commercial sources. Based on these criteria a preliminary screening of miRNAs present in soy and corn was conducted. Analysis of soy and corn RNA detected the abundant amount of miR156a, -164a, and -167a, however, miR171j was not detected in either sample (Fig 2). Mouse liver and fecal RNA samples were also analyzed for zma-miR171j, and no amplification was detected. Soy and mouse miRNAs were searched for sequence homogeneity to zma-miR171j in miRBase (Release 21), and no exact match was found. Therefore, corn miRNA zma-miR171j was selected as the internal control for our assay development/improvement below. The linear range for qRT-PCR detection was then constructed and synthetic miR171j from 0.005 amol to 500 fmol was used. Concentrations ranging from 0.016 amol to 250 fmol were determined to be within the linear range (Ct = 6.808 to 32.151, R^2^ = 0.997). Given their differential sensitivity toward degradation, both methylated and unmethylated miR171j (Table 1) were synthesized by Integrated DNA Technologies (Coralville, IA) and used in the assay development as positive (unmethylated) and negative (methylated) controls.

### Comparison of periodate oxidation and β elimination methods using internal controls

**Fig 3:**
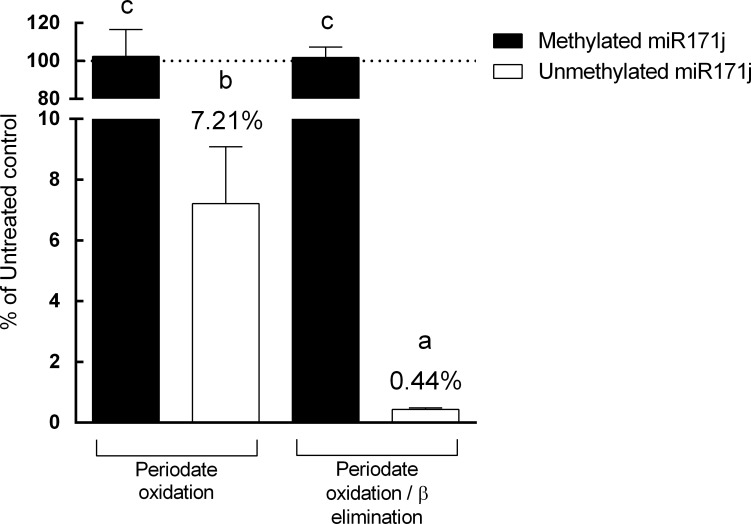
Comparison of periodate oxidation and β elimination methods. 20 ng of methylated miR171j or unmethylated miR171j were treated with 1) periodate oxidation, or 2) periodate oxidation followed by β elimination, and analyzed by qRT-PCR as described in Materials and Methods. Data is presented as a percentage of miR171j detected in the treatment group to that of the control group. Columns marked with different letters are significantly different from each other (*p* ≤ 0.05, data presented as Mean ± SD).

In previous reports, periodate oxidation has been used alone or followed by β elimination [8, 15, 36]. The efficiency of these two methods was not clear and was compared in this study using the internal controls designed above. Twenty ng of synthetic methylated miR171j and unmethylated miR171j were 1) treated with periodate for 40 min on ice or 2) treated with periodate for 60 min at room temperature, followed by termination of periodate reaction and β elimination with NaOH for 60 min, which were two commonly employed protocol conditions in the literature [8, 15, 36]. After periodate oxidation, no significant degradation (*p* = 0.62) was observed in methylated miR171j, and 92.79% unmethylated miR171j was degraded (*p*<0.0001) (Fig 3). When both periodate oxidation and β elimination were used, no significant degradation was observed in methylated miR171j (*p* = 0.62), and over 99% of the unmethylated miR171j was degraded (*p*<0.0001) (Fig 3). Therefore, all of the following experiments used the combination of periodate oxidation and β elimination.

**Fig 4:**
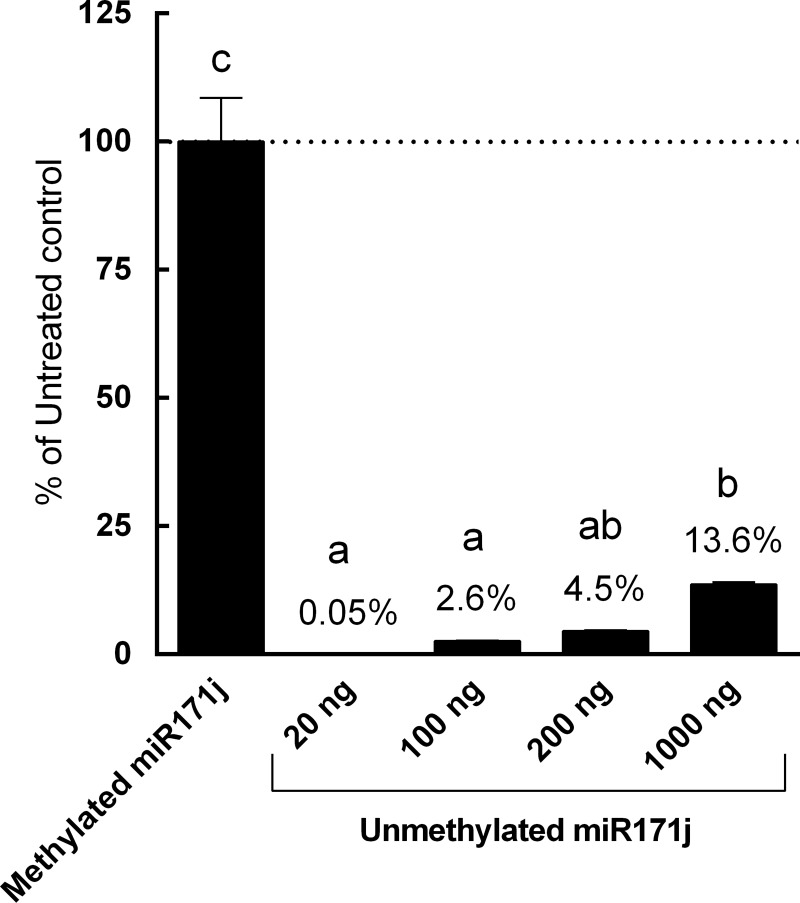
Concentration-dependent decrease in treatment efficiency of unmethylated miRNA. 20 ng of methylated miR171j or 20–1000 ng of unmethylated miR171j were treated with or without periodate, followed by β elimination, and analyzed by qRT-PCR as described in Materials and Methods. Data is presented as a percentage of miR171j detected in the treatment group to that of the control group. Columns marked with different letters are significantly different from each other (*p* ≤ 0.05, data presented as Mean ± SD).

The capacity of the assay system was then tested using varying amounts (20 to 2000 ng) of unmethylated miR171j in a fixed assay volume (100 μL) under the periodate oxidation/β elimination protocol described above. As the concentration of miR171j increased, the percentage of degradation significantly decreased (Fig 4). 2000 ng miR171j in 100 μL exceeded the linear range of detection using qRT-PCR (Ct < 6.808). At 1000 ng, 13.6% of unmethylated miR171j remained in the reaction system, which was significantly more than concentrations lower than 200 ng (*p*<0.0001).

### Detection of selected miRNA in plant RNA matrix

**Fig 5:**
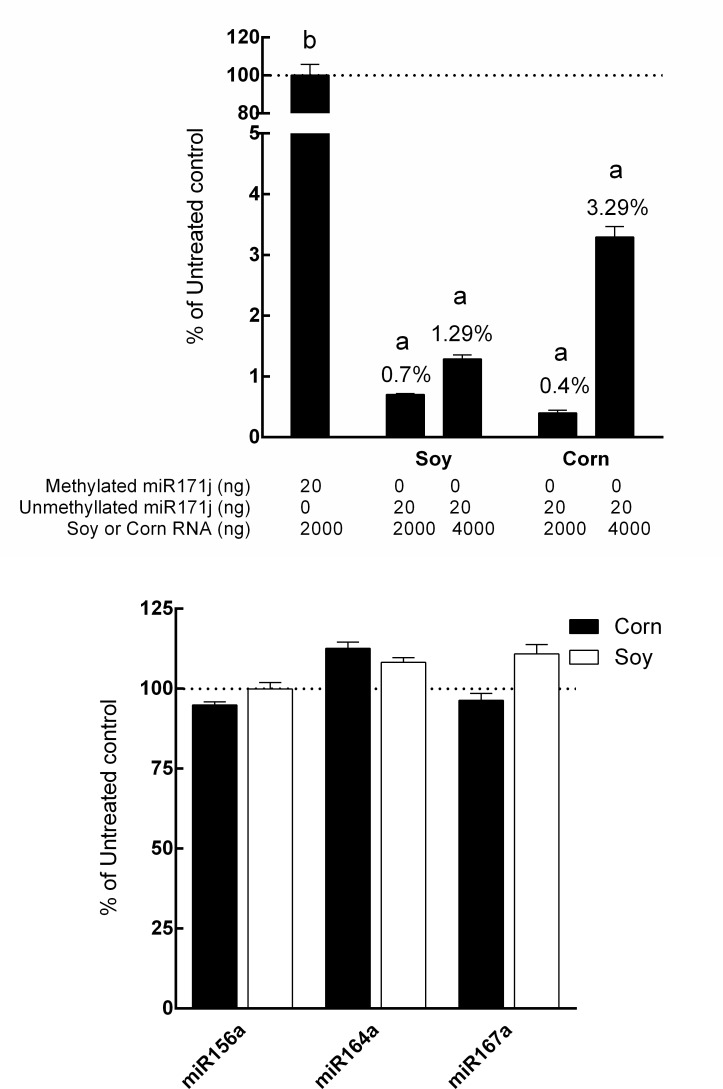
Evaluation of internal control in plant RNA matrix and plant miRNAs. A) 20 ng of methylated or unmethylated miR171j added to 2000 or 4000 ng of soy or corn total RNA or B) 2000 ng corn and soy total RNA were treated with or without periodate, followed by β elimination, and analyzed by qRT-PCR as described in Materials and Methods. Data is presented as a percentage of miRNAs detected in the treatment group to that of the control group. Columns marked with different letters are significantly different from each other (*p* ≤ 0.05, data presented as Mean ± SD).

Abundant miRNAs (miR156a, -164a, and -167a) in soy and corn were analyzed using qRT-PCR with methylated and unmethylated miR171j (20 ng/assay) included as internal controls in 2 μg or 4 μg plant RNA matrix. After treatment with the periodate oxidation/β elimination protocol, over 99% of unmethylated miR171j was degraded in both soy and corn matrices at 2 μg (Fig 5). There was no significant degradation of methylated miR171 (*p* = 0.82). At 4 μg, there appeared to be a slight drop in efficiency especially in corn to ~ 96% degraded (Fig 5). There were no changes in the amount of miR156a, -164a, and -167a levels before and after periodate oxidation/β-elimination treatment (Fig 5). The amounts of the selected miRNAs analyzed in corn and soy samples are shown in Table 3. The miR167a appeared to be the most abundant among the three tested in soy and corn. By contrast, miR164a appeared to be the least abundant.

**Table 3:** miRNA concentration in corn and soy.

	Corn (pg/g of fresh corn kernel)	Soy (pg/g of dry soybean)	Chow (pg/g of diet)
miR156a	3.32	6.22	13.1
miR164a	2.78	0.23	1.31
miR167a	56.65	20.93	143.9

### Evaluation of internal controls in animal RNA matrix

**Fig 6:**
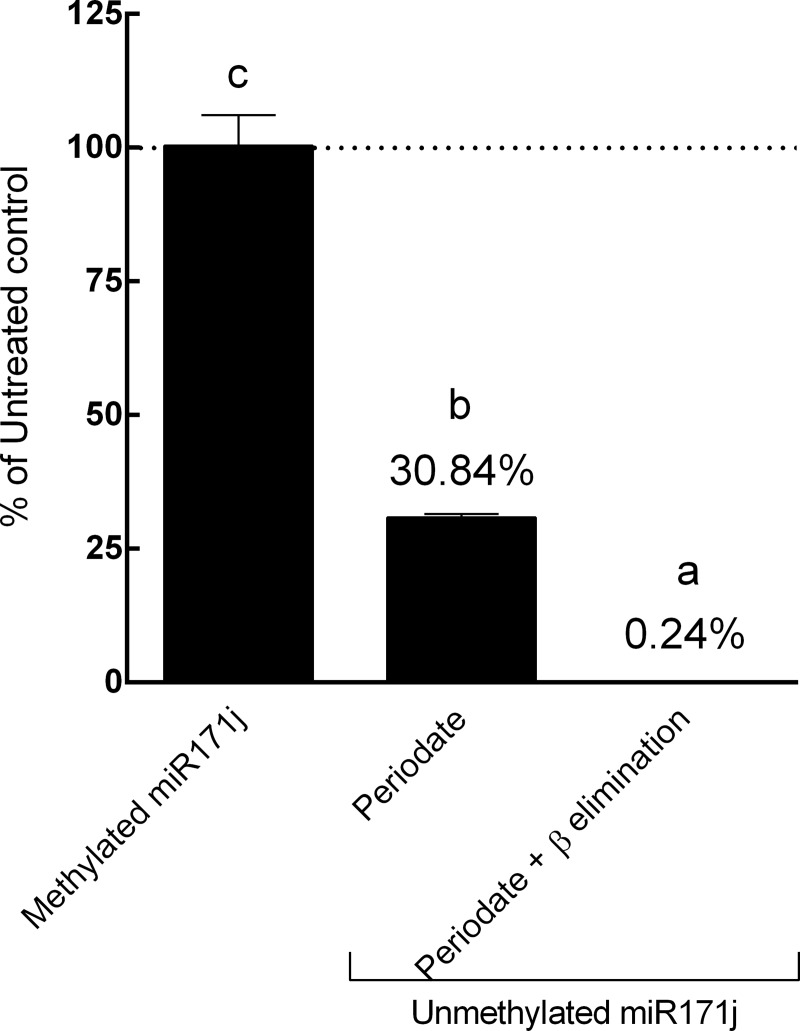
Comparison of periodate oxidation and β elimination methods in the liver matrix. 20 ng of methylated miR171j or 20 ng of unmethylated miR171j were added to 2000 ng of mouse liver total RNA treated with periodate alone, or followed by β elimination, and analyzed by qRT-PCR as described in Materials and Methods. Data is presented as a percentage of miR171j detected in the treatment group to that of the control group. Columns marked with different letters are significantly different from each other (*p* ≤ 0.05, data presented as Mean ± SD).

**Fig 7:**
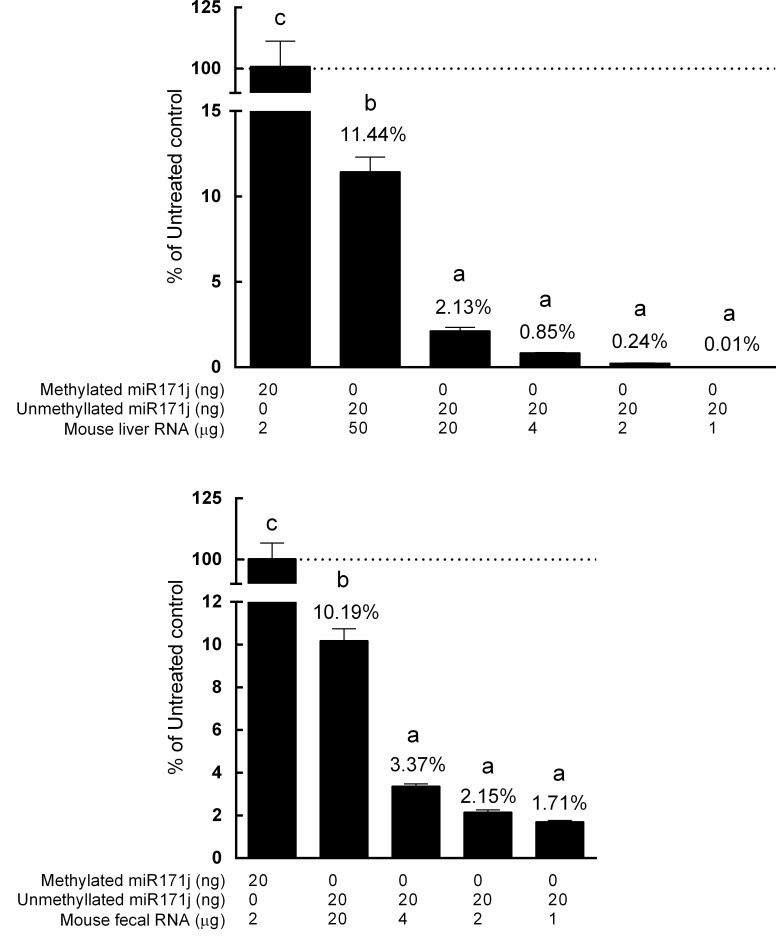
Evaluation of internal control in mammalian RNA matrix. 20 ng of methylated miR171j or 20 ng of unmethylated miR171j were added to A) 1 μg to 50 μg of mouse liver total RNA or B) 1 μg to 20 μg of mouse fecal total RNA treated with or without periodate, followed by β elimination, and analyzed by qRT-PCR as described in Materials and Methods. Data is presented as a percentage of miR171j detected in the treatment group to that of the control group. Columns marked with different letters are significantly different from each other (*p* ≤ 0.05, data presented as Mean ± SD).

Twenty ng of methylated or unmethylated miR171j were spiked into 2 μg total RNA isolated from mouse liver. When treated with periodate oxidation alone, only 70% unmethylated miR171j was degraded, leaving a significant amount of unmethylated miR171j intact. More importantly, periodate treatment followed by β elimination degraded >99% unmethylated miR171j (*p*<0.0001) (Fig 6). To test the limit of periodate treatment and β elimination, 1 to 50 μg liver RNA was used. As the amount of liver RNA increased, the percentage of degradation in the unmethylated miR171j significantly decreased (Fig 7). In 1 to 20 μg liver RNA, no significant decrease of degradation of unmethylated miR171j was detected, however, in 50 μg liver RNA, 11.44% unmethylated miR171j was left intact after periodate oxidation and β elimination (*p*<0.0001). A similar decrease of degradation efficacy was also observed in treatment using fecal RNA as a matrix. No significant decrease of degradation of unmethylated miR171j was detected between 1 to 4 μg fecal RNA (Fig 7). In 20 μg fecal RNA, significantly higher percentage (10.19%) of unmethylated miR171j was detected (*p*<0.0001) (Fig 7).

### Detection of plant miRNA in animal diets

miR156a, -164a, and -167a were detected in abundant amount in corn/soy-based chow diet. After periodate oxidation/β elimination, 30.6%, 38.4%, and 23.2% of miR156a, -164a, and -167a in chow diet were determined to be in methylated forms, which were calculated to be 2.0, 0.19, 21.4 fmol/g of diet, respectively. Analysis of purified AIN-93M rodent diet detected miR156a, -164a, and -167a at Ct of 30.83 ± 0.37, 34.02 ± 0.22, and 29.66 ± 1.36 cycles, respectively. However, after periodate oxidation/β elimination, there was no difference between diet and no-template controls.

### Detection of plant miRNA in plasma, fecal and liver samples

To determine whether the matrix interferes with the qPCR detection of plant miRNAs when analyzing mouse samples, synthetic miR156a, 164a, and 167a were spiked in a mouse liver RNA matrix and analyzed. Synthetic miRNAs (0.5–10 pg/μL) were added to 20 ng/μL liver RNA matrix, and compared to the standard curves. No difference in qRT-PCR cycle number was observed. Analyses of plasma and fecal samples collected from animals fed with chow diet demonstrated that the Ct value was not different from no-template control even before periodate oxidation/β elimination (Table 4). In liver, miR156a, -164a, and -167a were detected at 34.18 ± 0.73, 34.06 ± 0.41, and 32.47 ± 1.15 cycles in qRT-PCR, respectively. Though out of the linear detection range, these cycles were significantly lower based on t-test than those of the no-template controls’ 35.19 ± 0.34, 35.19 ± 0.76 and 34.76 ± 0.67 cycles, respectively (Table 5). However, after periodate oxidation and β elimination, miR156a and -167a required more cycles for detection, 36.07 ± 1.35 and 34.24 ± 1.02 cycles, respectively which were not statistically different from the number of cycles of the no template controls in a Tukey’s test.

**Table 4:** Plasma and fecal miRNA amplification cycle numbers.

	Plasma		Feces	
	Before treatment	No-template control	Before treatment	No-template control
miR156a	31.67 ± 0.21	31.60 ± 0.04	33.62 ± 0.64	33.69 ± 0.14
miR164a	33.90 ± 0.38	33.93 ± 0.24	30.85 ± 0.37	30.72 ± 0.01
miR167a	32.65 ± 0.55	32.58 ± 0.14	31.11 ± 1.30	30.19 ± 0.07

Cycle numbers were averages of plasma or fecal samples (n = 5) or no-template controls (n = 3). No significant differences were detected between before treatment and no-template control in a t-test (*p*>0.1).

**Table 5:** Liver miRNA amplification cycle numbers.

	Before treatment	After treatment	No-template control	t-test *p* value
miR156a	34.18 ± 0.73 ^a^	36.07 ± 1.35 ^b^	35.19 ± 0.34 * ^ab^	0.02
miR164a	34.06 ± 0.41 ^a^	35.06 ± 0.67 ^a^	35.19 ± 0.76 * ^a^	0.03
miR167a	32.47 ± 1.15 ^a^	34.24 ± 1.02 ^b^	34.76 ± 0.67 * ^b^	0.02

Cycle numbers were averages of liver samples (n = 5) or no-template controls (n = 3). Asterisks represent significant differences between before treatment and no-template control in a t-test. Numbers with different letters are significantly different in a Tukey’s test (*p* ≤ 0.05) among the three groups.

## Discussion

In this study, we presented a modified/refined method (see S1 File for the detailed protocol) of detecting plant miRNA in different biological matrices using qRT-PCR. Importantly, the inclusion of the alkaline β-elimination step was shown to significantly improve the efficacy of degradation of unmethylated miRNAs. Additionally, internal controls (miR171j) were designed and incorporated into the assay that allows for monitoring completion of the degradation reaction through periodate oxidation and β elimination. As demonstrated in this study, the inclusion of internal standards and modification of the protocol improved our confidence in the validity of plant miRNA detection in a wide range of matrices, consistent with that recently proposed in a review of miRNA analysis and methodological considerations [26].

Using the synthesized methylated and unmethylated miRNAs, we were able to validate the efficacies of periodate oxidation and β elimination. Previous reports used periodate oxidation alone or followed by β elimination to react with the unmethylated 3’ end of miRNAs [8, 15, 22, 36]. The 2’,3’-hydroxy termini on unmethylated miRNA, which is oxidized by periodate, cannot be ligated to sequencing adapters and thus are prevented from being sequenced [20, 21, 37]. Therefore, skipping β elimination when using sequencing as the detection method may bear less impact.

Currently, plant miRNA qRT-PCR assays are designed based on a previous publication [38] using stem-loop reverse transcription primers, which were widely used in previous analyses of miRNAs [8, 11, 15, 16, 22, 39]. The stem-loop reverse transcription primer for cDNA synthesis used qRT-PCR detection method can specifically recognize the 3’ end of miRNA, however, does not involve ligation. Therefore, periodate oxidation alone may not be sufficient to affect the hybridization between the stem-loop reverse transcription primer and target miRNA. Our comparison of periodate oxidation alone and periodate oxidation followed by β elimination demonstrated that the combination was necessary (Fig 4). Furthermore, another benefit of β elimination is that it may also eliminate the pri- and pre-miRNAs being processed in the cell. Pri- and pre-miRNAs contain the same sequences as the mature counterpart [40], since the stem-loop primer is specific only to the 3’ end, regardless of the methylation status and 5’ end sequence. Thus, incompletely edited pri- or pre-miRNA or immature miRNA:miRNA* duplex may be reverse transcribed into complementary DNA and amplified by qRT-PCR. β elimination step can degrade such immature miRNAs, and therefore, when qRT-PCR is used as the detection method, β elimination is an essential step for miRNA degradation.

Periodate is a powerful oxidizing agent, and the 2’-, 3’- terminal-*cis*-diol group at 3’ end of unmethylated miRNA can be oxidized by periodate to dialdehyde, and subsequently removed by β elimination [24]. Our study showed that the efficiencies of oxidation and degradation treatments significantly decreased when high concentration (>1000 ng) of unmethylated miRNA was introduced into the reaction system (Fig 4). These results indicate that overloading the system may influence the detection of mature miRNA and lead to overestimation or a false positive in a sample when using sequencing or qRT-PCR as the detection method. However, the inclusion of internal standards such as described herein allows for the monitoring of reaction efficiency and provides a more accurate quantitation of plant miRNA in samples. Although not tested here, the inclusion of internal standards may also improve detection using sequencing.

Use of internal standards also allows us to test the capacity of the detection assay using mammalian samples. Mammalian miRNAs do not have 3’ methylation [20, 21], and will compete with the spike-in unmethylated miR171j for the oxidation power of periodate. In the literature, for detection of plant miRNA, a range of 20 to 100 μg RNA in 100 μL in a periodate treatment has been reported [8, 36]. In our study, RNA concentrations higher than 20 μg led to a significant amount of un-degraded unmethylated miR171j internal control detected in the reaction system (Fig 7), indicating the capacity of the assay was overwhelmed. This is consistent with a previous study using gel electrophoresis which showed multiple bands (original miRNA and miRNA eliminated of 3’ end nucleotide) after periodate treatment of 100 μg RNA in 100 μL [36], indicating incomplete degradation. Using periodate oxidation alone for 40 min, without β elimination, left 30% unmethylated miR171j intact in a 2000 ng liver RNA matrix. These results indicate that the treatment was ineffective for differentiating the methylation status of miRNA samples, and quantitative analyses of plant miRNA in mammalian by qRT-PCR may not be accurate. Hence, the inclusion of internal standards as well as not overloading the assay system will greatly improve the validity and accuracy of plant miRNA measurement in different matrices.

Several periodate oxidation/β-elimination resistant plant miRNAs (miR156a, -164a, and -167a) from corn and soy were detected in the chow diet in comparable amounts to or higher than those in the chow diet used in Zhang’s study [8]. Our analysis of the mouse liver samples from animals fed with chow detected plant miRNA at Ct values of 32 to 34 cycles (Table 5) without periodate oxidation/β-elimination treatments. When these samples were treated with periodate, no detectable levels of miR156a, -164a, and -167a in plasma or liver were found. The false detection in liver samples without periodate oxidation/β-elimination treatment was likely a result of non-specific amplification at high cycle numbers [41]. Therefore, sample treatment with periodate oxidation/β-elimination is necessary to avoid false positives. Furthermore, based on our analysis, plant miRNA was not present in the liver of mice fed corn/soy-based chow diet.

The inclusion of spike-in methylated and unmethylated miRNAs as internal controls has two important advantages: 1) Unmethylated miRNA internal control can be used for controlling the efficiency of degradation. A previous study used endogenous mammalian miRNA as a control for periodate treatment efficiency [8], however, endogenous miRNAs vary in concentrations. One can achieve higher concentrations than endogenous miRNAs by using a spike-in control, and the complete degradation of spike-in control provide information to indicate complete degradation of all endogenous miRNAs. Previous research reported that mammalian RNA contains less than 0.1% of miRNA [42], and 1% of internal control was used in this study to ensure that it in excess of the endogenous miRNAs. 2) Methylated miRNA internal control can be used to adjust for the efficiency of RNA isolation and purification. A similar argument was made in previous reports showing that under-estimation of miRNA concentration could have resulted from loss during RNA isolation [22]. Periodate oxidation and β elimination treatment of miRNA involves multiple steps of RNA isolation and purification, therefore, the inclusion of methylated miRNA internal control can help control the efficiency of RNA isolation and purification. We further propose that both unmethylated and methylated miRNAs need to be used in the same treatment as positive and negative internal controls to adjust for the efficiencies of both periodate oxidation/β-elimination treatment and RNA isolation recovery.

Following the first report of cross-kingdom miRNA transfer in 2012 [8], analysis of sequencing data in human plasma expanded the list of sources of exogenous miRNAs detected in human plasma to include plant, bacteria, and fungi [13, 14], and more plant miRNAs (miR159 and miR2911) were reported to be identified in human serum [15, 16]. On the other hand, Dickinson and colleagues repeated Zhang’s animal feeding study with rice diet and were unable to detect rice miRNAs in plasma and liver [11]. Another study searched for plant miRNA sequences in public sRNA datasets and determined that miR168 was extremely over-represented in animal sRNA datasets, which may result from contamination in the process of sequencing [9, 17]. Further analyses in human and mouse after oral consumption of a diet containing plant miRNAs found no detectable level of miRNA uptake in plasma or liver [10, 12, 18]. Feeding studies in insects also found no plant miRNA in insect carcass [9, 10]. These studies highlight a major obstacle in assessing the origin of miRNAs in biological samples is the difficulty in differentiating plant miRNAs from other species such as mammalian miRNA. The protocol developed in this study, we believe, provides a remedy to this problem.

In this study, we showed the importance of using periodate oxidation/β elimination treatment, an appropriate amount of RNA, and including internal controls to improve the specificity and accuracy of quantification of mature plant miRNA in biological matrices. Considering the discrepancy in the literature for detection of exogenous miRNA in mammalian plasma and tissue, these simple modifications allow for better confirmation or rejection of the novel idea of cross-kingdom miRNA transfer.

## Supporting information

S1 File Protocol. Experimental protocols of periodate oxidation and periodate oxidation with β-elimination.
(DOCX)
