# Supplementary material for: An improved method to quantitate mature plant microRNA in biological matrices using modified periodate treatment and inclusion of internal controls
Source: PLoS One. 2017 Apr 11;12(4):e0175429. doi: 10.1371/journal.pone.0175429 (PMC5388493; doi:10.1371/journal.pone.0175429)
Supplement: S1 File — Experimental protocols of periodate oxidation and periodate oxidation with β-elimination. (DOCX) [file pone.0175429.s001.docx]

**Protocol**

Periodate oxidation

1. A reaction mix consisting of 95 μl small RNA and 5 µl 200 mM sodium periodate (freshly prepared) was incubated for 40 min in the dark at 0 °C.
2. 1 µl glycogen, 10 µl 3 M sodium acetate (pH 5.2, DEPC-treated), and 300 µl ethanol was added to the reaction mix.
3. The mixture was incubated in -20 °C for 20 min and centrifuged at 20,000 g at 4 °C for 10 min to precipitate RNA.
4. RNA was washed with 70% ethanol twice, and supernatant was aspirated.
5. RNA was dissolved in nuclease-free water and kept at -20 °C.

Periodate oxidation and β-elimination

1. An 87.5 µl reaction mix consisting of small RNA in 0.06 M borax/boric acid buffer (pH 8.6) was prepared and 12.5 µl of 200 mM sodium periodate (freshly prepared) was added to the reaction mix.
2. The reaction mix was incubated in the dark at room temperature for 1 h.
3. 10 µl of glycerol was added to the reaction mix and incubated for another 30 min to stop the reaction.
4. 1 µl glycogen, 10 µl 3 M sodium acetate (pH 5.2, DEPC-treated), and 300 µl ethanol was added to the reaction mix.
5. The mixture was incubated in -20 °C for 20 min and centrifuged at 20,000 g at 4 °C for 10 min to precipitate RNA.
6. RNA was washed with 70% ethanol twice, and supernatant was aspirated.
7. Precipitated RNA was dissolved in 100 µl of 0.055 M borax/boric acid/NaOH (pH 9.5) and incubated for 90 min at 45°C.
8. 1 µl glycogen, 10 µl sodium acetate, and 300 µl ethanol was added to the reaction mix.
9. The mixture was incubated in -20 °C for 20 min and centrifuged 20,000 g at 4 °C for 10 min to precipitate RNA.
10. RNA was washed with 70% ethanol twice, and supernatant was aspirated.
11. RNA was dissolved in nuclease-free water and kept at -20 °C.
